# A legal judgment prediction model based on knowledge fusion and dependency masking

**DOI:** 10.1371/journal.pone.0340717

**Published:** 2026-01-16

**Authors:** Yishan Chen, Xiaoyi Zhu, Zhiyun Zeng, Pengfei Wang, Xinhua Zhu

**Affiliations:** 1 School of Business, Guilin Tourism University, Guilin, China; 2 School of Computer Science and Technology, China University of Mining and Technology, Xuzhou, China; 3 School of Computer Science and Engineering, Guangxi Normal University, Guilin, China; 4 School of Electrical and Electronic Engineering, Nanyang Technological University, Singapore; Beijing Institute of Technology, CHINA

## Abstract

Legal Judgment Prediction (LJP) is a core task in Legal AI systems, which aims to predict law articles, charges, and term-of-penalty from case facts. While existing deep-learning-based LJP approaches for civil law systems have achieved certain progress, they still suffer from two key limitations: (1) insufficient deep understanding and effective utilization of external judicial knowledge; and (2) the lack of effective strategies to filter out erroneous dependency information in multi-task LJP frameworks. To address these challenges, we propose a legal judgment prediction model based on knowledge fusion and dependency masking. Specifically, we first integrate a CNN-based local semantic refinement component into the existing BERT-based legal knowledge extraction method, thereby enabling the model to further extract the core knowledge embedded in judicial documents. Then, we introduce differential attention to reduce noise in conventional attention fusion methods and help the model locate key information in case facts more accurately. Furthermore, we propose a multi-task dependency information masking mechanism to accurately identify and filter erroneous dependency information for multi-task LJP methods. Experiments conducted on real-world datasets demonstrate the superiority of our proposed model. This code is available online at https://github.com/PaperCode-GNU/KFTM.

## 1. Introduction

With the rapid development of neural networks and deep learning technologies, natural language processing (NLP) has been increasingly applied in various fields, which also plays a crucial role in building legal artificial intelligence (legal AI). From a practical application perspective, legal AI systems have demonstrated notable positive effects in multiple scenarios [1–3]. Firstly, they can effectively ease the heavy workload of legal professionals, enabling these professionals to devote more energy to key legal matters. Secondly, for judicial personnel, legal AI systems can serve as a powerful auxiliary tool, helping them analyze case situations more efficiently and retrieve relevant case information with precision, thereby providing a more sufficient basis for case handling. Furthermore, legal AI systems can offer timely and necessary assistance to those in need of legal aid, effectively reducing the cost of accessing legal services while increasing their chances of obtaining judicial assistance—ultimately promoting the fair distribution of judicial resources on a broader scale.

**Fig 1:**
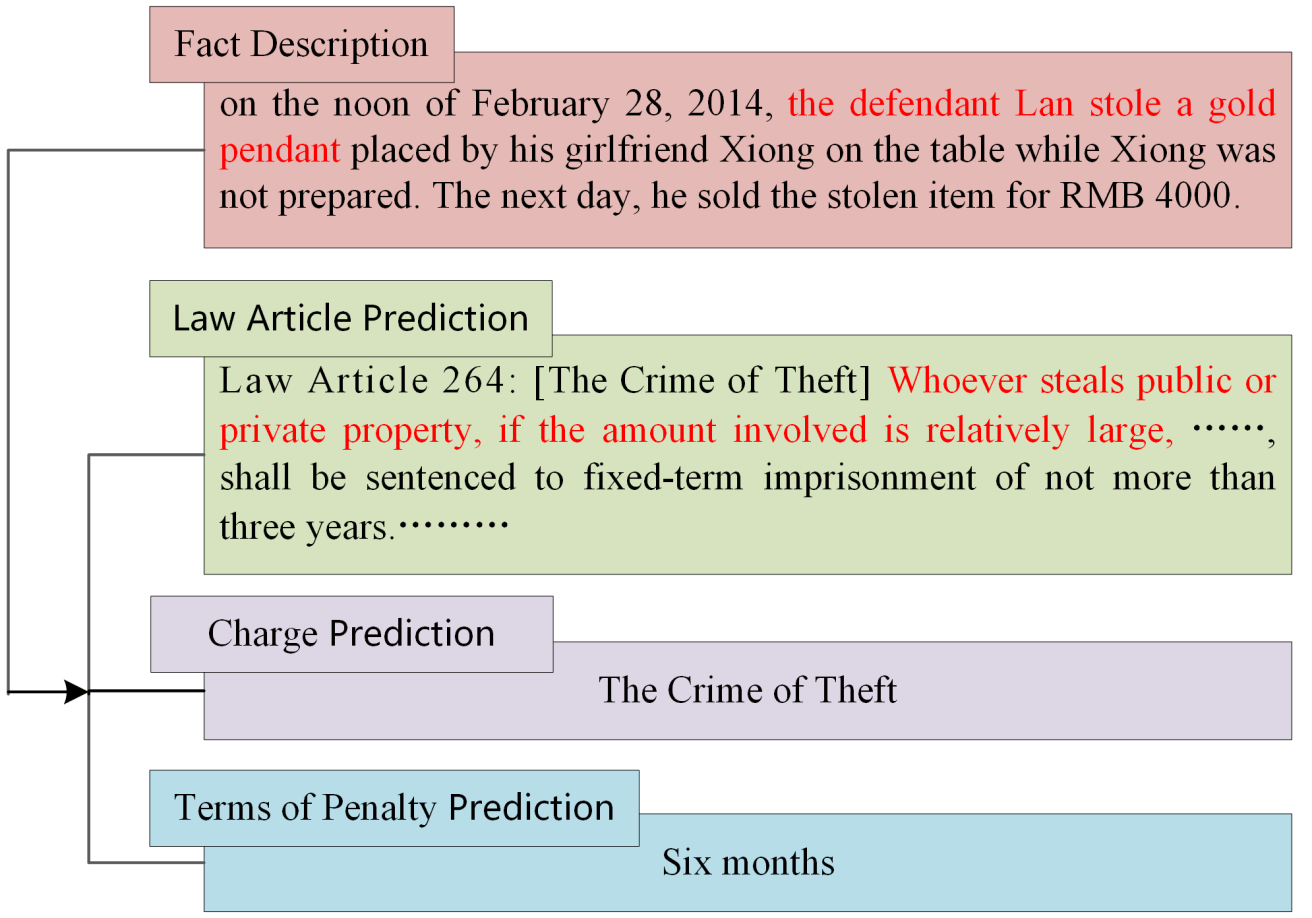
A examples of legal judgment prediction.

Legal judgment prediction (LJP) is a key task in the Legal AI system for civil law systems, which aims to predict the law articles, charges, and term of penalty based on case facts [2], as shown in Fig 1. Existing deep-learning-based LJP methods in civil law systems are categorized into two types [3]: single-task models and Multi-Task Learning (MTL) methods. The former typically focuses on optimizing the network architectures [4,5] or incorporating additional information sources [6,7] for specific subtasks. The latter treats multiple subtasks as an integrated whole and adopts the MTL framework for unified modeling. This has made MTL the current mainstream approach, given that the LJP in civil law systems usually encompasses several interrelated subtasks. The technical route of MTL aims to design different decoding structures for each subtask based on case facts, primarily focusing on the following four aspects: (1) Conventional MIT methods for parameter sharing [8], which focus on how to share parameters among related subtasks to improve the generalization ability of the model. (2) Dependency-based MIT methods [9–11], which incorporate the dependency relationships between subtasks into the MIT framework to improve the prediction accuracy. (3) MIT methods with knowledge fusion [12–14], whose goal is to strengthen the representation of case facts through legal knowledge. (4) Enhanced MIT methods, which improve the model’s discrimination ability through contrastive learning [3] or graph neural networks [15,16].

Currently, although MTL-based legal judgment prediction methods have made certain progress, they still have the following limitations: First, they lack in-depth understanding and exploration of the judicial knowledge contained in officially issued legal documents, as well as research on how to leverage such judicial knowledge to strengthen the representation of case facts. Second, in dependency-based MTL frameworks, there is a lack of effective strategies to filter out erroneous information in dependencies, thereby reducing the negative impact of such erroneous dependencies on subsequent tasks.

To address the first issue mentioned above, we first systematically collate law articles and charge interpretations corresponding to the corpus labels from the Criminal Law of the People’s Republic of China. We then extract judicial knowledge from these legal texts using the Linguistically-motivated bidirectional Encoder Representation from Transformer (LERT) [17] and Convolutional Neural Networks (CNNs), and fuse the extracted judicial knowledge into case fact representations through multi-head differential attention [18] to enhance their semantic expressiveness. Regarding the second issue, we propose a multi-task dependency information masking mechanism. This mechanism determines whether to mask task dependency information by judging the correctness of the prediction results from preceding tasks, thereby accurately identifying and filtering erroneous dependency information. The main contributions of this study are summarized as follows:

(1) We propose a legal knowledge extraction module that integrates a CNN-based local semantic refinement component into the existing BERT-based legal knowledge extraction method [14]. This enables the model to further extract the core knowledge embedded in judicial documents.
(2) We propose a novel approach for fusing external judicial knowledge into case fact representations, which introduces differential attention [18] to reduce noise in existing attention-based fusion methods [12–14] and help the model locate key information in case facts more accurately.
(3) We propose a multi-task dependency information masking mechanism to accurately identify and filter erroneous dependency information in dependency-based MIT methods [9–11].

The remainder of this paper is organized as follows: Section 2 summarizes and details related works. Section 3 proposes a legal judgment prediction model based on knowledge fusion and dependency masking. Section 4 describes the evaluated datasets and experimental settings and presents the experimental results. Finally, Section 5 concludes the paper.

## 2. Related works

In the early stages, researchers commonly used classical machine learning methods to implement LJP tasks. For example, Şulea et al. [19] used Support Vector Machine (SVM) technology to process and mine the hidden key information in legal text data. Katz et al. [20] constructed a time-evolving Random Forest classifier prediction model, which only uses available data and unique feature engineering before the judgment decision is made to predict case judgment results. These early machine learning methods had obvious limitations. On the one hand, when analyzing and processing legal data, these methods struggle to mine deep-level text feature information. On the other hand, when handling LJP tasks, they fail to fully attach importance to the close internal connections between subtasks.

With the rapid development of deep learning (DL) technology based on multi-layer neural networks, researchers have applied techniques related to LJP tasks. Multi-layer neural networks have an excellent ability to learn complex knowledge and feature representation automatically from massive data, which can better cope with the complex, diverse, and semantically rich characteristics of legal text data. For example, Wei et al. [21] used CNNs for text classification in legal document review and achieved better classification results than SVM. Luo et al. [22] introduced an attention mechanism in stacked neural networks to predict charges with legal basis, which has a good generalization.

Existing deep-learning-based LJP methods in civil law systems mainly focus on the following three aspects for research: (1) Deep exploration and utilization of the dependency relationships between subtasks to improve classification accuracy. For example, Zhong et al. [9] proposed a TopJudge model that considers unidirectional topological dependencies between subtasks and models their explicit dependencies with a scalable Directed Acyclic Graph (DAG). Yang et al. [10] constructed a dual-feedback framework involving multi-perspective forward prediction and backward verification, and proposed an MPBFN model that considers bidirectional topological dependencies. Zhang et al. [11] employed consistency and distinction distillation to model label topological relation among multiple subtasks. (2) Fusing legal knowledge into case facts to enhance their semantic expressiveness [13,14]. Among them, neural networks such as CNNs and BERT [23] are used to extract legal knowledge; while attention mechanisms are used to fuse legal knowledge into case fact representations, helping the model locate key fact information that determines the judgment. (3) Using contrastive learning [3] or graph neural networks [15,16] to enhance the discriminative ability of the models. For example, Chen et al. [3] used case fact triplets for contrastive learning, which can enhance the model’s ability to distinguish the relevance and differences of case facts; Xu et al. [15] constructed a self-learning graph attention network to distinguish between confusing law articles and charges; and Dong et al. [16] used graph contrastive learning and data augmentation techniques to enhance the model’s ability to distinguish different situations.

Pre-trained language models are currently widely used as encoders for LJP tasks. There are two pre-training methods for Chinese language models. One approach is to integrate Chinese linguistics into BERT, such as Cui et al. [17] proposed LERT (Linguistically-motivated bidirectional Encoder Representation from Transformer), which is a BERT-based pre-trained model enhanced by three Chinese linguistic tasks: Part-of-Speech tagging, Named Entity Recognition, and Dependency Parsing. Zhang et al. [24] proposed CKBERT (Chinese knowledge-enhanced BERT), which used linguistic-aware masked language model and contrastive multi-hop relation model for pre-training. Another method is to integrate domain knowledge into BERT, and a well-known example is the Open Chinese Language Pretrained Model Zoo proposed by Zhong et al. [25], whose Legal BERT was applied as an encoder in [11] and [14]. Considering that LERT incorporates knowledge from Chinese encyclopedias and various linguistic disciplines, we have chosen it as the BERT encoder for this study in the hope of achieving a more comprehensive semantic understanding of Chinese texts.

The aforementioned deep learning-based LJP methods have made certain progress; however, they still lack in-depth understanding and utilization of external judicial knowledge, as well as effective strategies to filter out erroneous information in multi-task dependencies. To address these issues, this study proposes a legal judgment prediction model based on knowledge fusion and dependency masking

## 3. Methodology

### 3.1 Model structure

**Fig 2:**
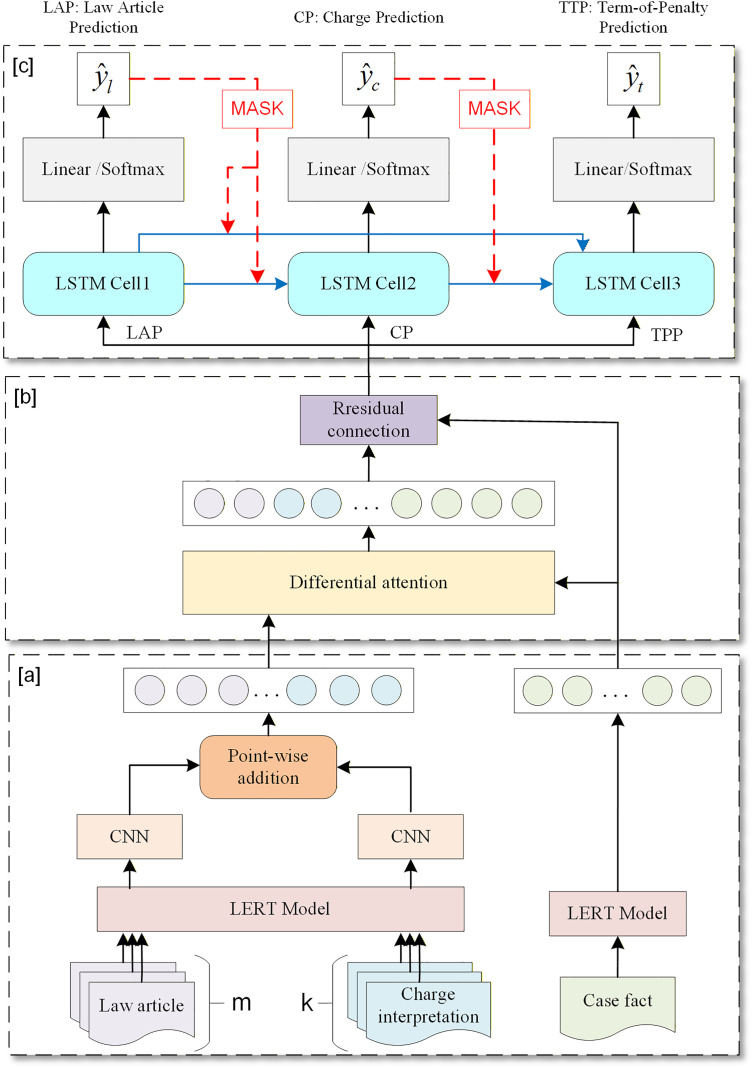
Proposed legal judgment prediction model structure.

The legal judgment prediction model proposed in this paper based on Knowledge Fusion and Dependency Masking is briefly referred to as KFDM, and its structure is shown in Fig 2. The KFDM model consists of three main modules, namely the Judicial Knowledge Extraction Module (JKEM) shown in Fig 2(a), Judicial Knowledge Fusion Module (JKFM) shown in Fig 2(b) and the Multi-task Dependency Masking Module (MDMM) shown in Fig 2(c).

In the process of extracting and fusing legal knowledge, accurately extracting the knowledge contained in legal documents and effectively eliminating the noise and deviation caused by confusing law articles and charges are challenging tasks. Our JKEM and JKFM are designed to solve these problems.

Our JKFM is composed of a pre-trained LERT model and a CNN module, which adds a CNN module for local semantic refinement on the existing BERT-based legal knowledge extraction method [14]. The advantage of BERT is that it deeply models global contextual dependencies such as cross-sentence reference and long-distance logical relationships through bidirectional and multi-layer Transformers, but it may not pay enough attention to local details. CNN can efficiently capture local continuous semantic units such as phrases, fixed collocations, and local grammatical structures through the window of convolutional kernels, thus forming a semantic complementarity with BERT that simultaneously covers global semantic logic and local key information [26,27].

After obtaining feature representations encompassing all law articles and charge interpretations, our JKFM employs a unique approach to fuse external judicial knowledge into case fact representations, which introduces differential attention to reduce noise in existing attention-based fusion methods [12–14]. Additionally, a residual connection is adopted to prevent the degradation of the original case feature caused by network depth and the incorporation of external judicial knowledge.

Dependency-based MIT methods [9–11] are widely adopted in LJP tasks. These methods aim to fully explore and utilize the inherent relationships between subtasks, thereby improving the performance of the overall model. However, in practical applications, it has been observed that although the dependency relationships between subtasks can provide additional information for the joint model, they also pose a non-negligible problem: namely, incorrect task dependency information may propagate in multi-task relationship modeling. To address this issue, our MDMM establishes a multi-task system with dependency masking for the three sub-tasks. During training, the model evaluates the prediction results of the preceding tasks: when predictions are accurate, task-dependency information is transmitted; when predictions are inaccurate, such dependency information is masked. This approach effectively identifies and masks incorrect task dependency information.

### 3.2 Legal knowledge feature extraction

(1) **Using LERT to process relevant legal texts**

We first extract the definition text of each law article label in the corpus from the Criminal Law of the People’s Republic of China to obtain the corresponding text content, forming a text set of law article $A=\{A_{\text{1}},A_{\text{2}},A_{\text{3}}\cdots ,A_{m}\}$ in the corpus. *m* is the number of law article labels in the corpus, and $A_{m}$ represents the text of the *m-th* law article in A. Then, we use the following formula to calculate the feature vector $v_{A}^{i}$ of the i-th legal clause $A_{i}$ in A:


$$\begin{array}{c} s_{token}^{A_{i}}=Tokenizer\left(A_{i}\right)\in R^{n_{A}} \end{array}$$ (1)

$$\begin{array}{c} v_{A}^{i}=LERT\left(s_{token}^{A_{i}}\right)=\left\{h_{\text{1}}^{A_{i}},h_{\text{2}}^{A_{i}},\cdots ,h_{n_{A}}^{A_{i}}\right\}\in R^{n_{A}\times d} \end{array}$$ (2)

where Tokenizer (•) represents the tokenizer of the LERT pre trained language model, $s_{token}^{A_{i}}$ is the token sequence of $A_{i}$ obtained after being processed by the tokenizer, $n_{A}$ represents the fixed length of the text of law articles in the Criminal Law, LERT (•) represents the pre-trained language model LERT, and d  is the dimension of hidden states in the LERT model. Continuing to extract features from all law article texts in set A, we can obtain the feature representation combination $v_{A}$ for all law articles as follows:


$$\begin{array}{c} v_{A}=\left\{v_{A}^{\text{1}},\;v_{A}^{\text{2}},\;v_{A}^{\text{3}},\cdots ,v_{A}^{m}\right\}\in R^{m\times n_{A}\times d} \end{array}$$ (3)

Furthermore, we extract the interpretative text of each charge label in the corpus from the Criminal Law of the People’s Republic of China, and process them similarly to the law articles to obtain the corresponding feature representation combination of the charge interpretation $v_{C}$:


$$\begin{array}{c} v_{C}=\left\{v_{C}^{\text{1}},\;v_{C}^{\text{2}},\;v_{C}^{\text{3}},\cdots ,v_{C}^{k}\right\}\in R^{k\times n_{C}\times d} \end{array}$$ (4)

where *k* is the number of charge labels in the corpus, $n_{C}$ represents the fixed length of the text of charges in Criminal Law.

(2) **Using CNNs for semantic refinement**

We use CNNs to further explore the main judicial knowledge contained within each law article and charge interpretation:


$$\begin{array}{c} \;\;\;{v_{A}^{i}}^{conv}=Conv\left(v_{A}^{i},W_{conv\_A}^{i},b_{conv\_A}^{i}\right)\in R^{n_{A}\times \frac{d}{\text{3}}} \end{array}$$ (5)

$$\begin{array}{c} \;{v_{C}^{i}}^{conv}=Conv\left(v_{C}^{i},W_{conv\_C}^{i},b_{conv\_C}^{i}\right)\in R^{n_{C}\times \frac{d}{\text{3}}} \end{array}$$ (6)

where ${v_{A}^{i}}^{conv}and\;{v_{C}^{i}}^{conv}$respectively represent the processing results of the *i*-th law article and charge interpretation representation $v_{A}^{i}$ and $v_{C}^{i}$ in CNNs, respectively. $W_{conv\_A}^{i}$ and $W_{conv\_C}^{i}$ respectively represent CNN convolution kernels used for extracting features from the law article and the charge interpretation, with a size of 3 × 3, an input channel of *d*, and an output channel of one-third of *d*. $b_{conv\_A}^{i}$ and $b_{conv\_C}^{i}$ are the deviations of two convolutions.

Then, we concatenate all the processing results to obtain the main law article and charge knowledge features $\hat{v_{A}}$ and $\hat{v_{C}}$:


$$\begin{array}{c} \hat{v_{A}}=\left[{v_{A}^{\text{1}}}^{conv};\;{v_{A}^{\text{2}}}^{conv};\cdots ;{v_{A}^{m}}^{conv}\right]\in R^{n_{A}\times \frac{md}{\text{3}}\;\;\;} \end{array}$$ (7)

$$\begin{array}{c} \hat{v_{C}}=\left[{v_{C}^{\text{1}}}^{conv};\;{v_{C}^{\text{2}}}^{conv};\cdots ;{v_{C}^{k}}^{conv}\right]\in R^{n_{C}\times \frac{kd}{\text{3}}\;}\; \end{array}$$ (8)

Furthermore, we apply linear transformations to map their dimensions back to *d*:


$$\begin{array}{c} \;\overset{―}{v_{\left\{A,C\right\}}}=\sigma \left(W_{\left\{A,C\right\}}\hat{v_{\left\{A,C\right\}}}+b^{\left\{A,C\right\}}\right)\in R^{{\{n}_{A}{,n}_{C}\}\times d} \end{array}$$ (9)

where $W_{\left\{A,C\right\}}\in R^{\frac{\left\{m,k\right\}d}{\text{3}}\;\;\times \text{d}}\;and\;b_{\left\{A,C\right\}}\in R^{d}$ are the weights and biases of the linear transformations, respectively; σ(·) represents the ReLU activation function.

Finally, the sequence lengths of $\overset{―}{v_{A}}$ and $\overset{―}{v_{C}}$ are uniformly extended to length $n_{S}$ by filling in zeros, and then they are added point by point to obtain a judicial knowledge feature representation $v_{S}$ containing law articles and charge interpretations:


$$\begin{array}{c} v_{S}=(\overset{―}{v_{A}}+\overset{―}{v_{C}})\in R^{n_{S}\times d} \end{array}$$ (10)

Linear processing for fusing the concatenation result $v_{S}$ is deferred to implementation in the subsequent differential attention calculation with case facts.

### 3.3 Legal knowledge fusion

**Fig 3:**
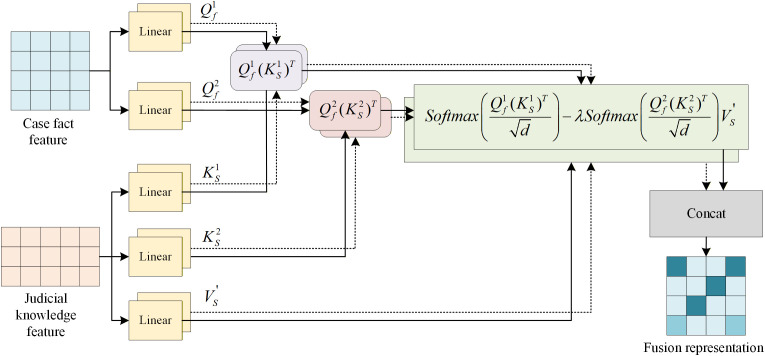
Proposed differential attention for fusing the judicial knowledge feature.

Let $v_{f}\in R^{n_{f}\times d}$ be a feature representation of a case fact to be processed in the pre-trained language model LERT, where $n_{f}$ is represents the length of the token sequence of the case fact. We apply differential attention [18] to fuse the judicial knowledge feature representation $v_{S}$ into the case fact feature representation $v_{f}$, as shown in Fig 3.

Firstly, we use linear layers to perform linear transformations on the fact feature representation $v_{f}$ and the judicial knowledge feature representation $v_{S}$, respectively, to prepare parameters for the differential scaled dot product attention. The linear transformation of the fact feature representation $v_{f}$ is used as the query Q for the attention, and the linear transformation of the judicial knowledge feature representation $v_{S}$ is used as the key K and value V for the attention. The specific calculation process is as follows:


$$\begin{array}{c} Q_{f}^{\text{1}}=v_{f}W_{Q}^{\text{1}}\in R^{n_{f}\times d} \end{array}$$ (11)

$$\begin{array}{c} Q_{f}^{\text{2}}=v_{f}W_{Q}^{\text{2}}\in R^{n_{f}\times d} \end{array}$$ (12)

$$\begin{array}{c} K_{S}^{\text{1}}=v_{S}W_{K}^{\text{1}}\in R^{n_{S}\times d} \end{array}$$ (13)

$$\begin{array}{c} K_{S}^{\text{2}}=v_{S}W_{K}^{\text{2}}\in R^{n_{S}\times d} \end{array}$$ (14)

$$\begin{array}{c} V_{S}^{\prime }=v_{S}W_{V}\in R^{n_{S}\times d}\; \end{array}$$ (15)

where $W_{Q}^{\text{1}}\in R^{d\times d}$ and $W_{K}^{\text{1}}\in R^{d\times d}$ are the linear transformation matrices of the query Q and key K in the first attention, respectively. $W_{Q}^{\text{2}}\in R^{d\times d}$ and $W_{K}^{\text{2}}\in R^{d\times d}$ are the linear transformation matrices of the query Q and key K in the second attention, respectively. $W_{V}\in R^{d\times d}$ is the linear transformation matrix of the value V shared by the two attentions.

We then calculate the difference between the two attentions and obtain the cross-attention difference result DiffAttn:


$$\begin{array}{c} DiffAttn=\left(softmax\left(\frac{Q_{f}^{\text{1}}{{(K}_{S}^{\text{1}})}^{T}}{\sqrt{d}}\right)-\lambda \;softmax\left(\frac{Q_{f}^{\text{2}}{{(K}_{S}^{\text{2}})}^{T}}{\sqrt{d}}\right)\right)V_{S}^{\prime } \end{array}$$ (16)

where λ is the hyperparameter. Referring to the method of Ye et al. [18], we set the hyperparameter λ as 0.2 to synchronize the learning dynamics.

To further enhance the expression effect of differential cross-attention, we use multiple heads to enhance the attention calculation and learn relevant judicial knowledge from different dimensions, and obtain the multi-head cross attention difference result $\;v_{mda}\in R^{n_{f}\times d}$:


$$\begin{array}{c} {head}_{i}={DiffAttn}_{i}\; \end{array}$$ (17)

$$\begin{array}{c} \overset{―}{{head}_{i}}=(\text{1}-\lambda )\cdot LN\left({head}_{i}\right) \end{array}$$ (18)

$$\begin{array}{c} V_{mda}=Concat\left(\overset{―}{{head}_{\text{1}}},\cdots ,\overset{―}{{head}_{i}}\right)W^{O} \end{array}$$ (19)

where $W^{O}\in R^{d\times \text{d}}$ is a learnable weight matrix, $\overset{―}{{head}_{i}}$ represents the attention value of the i-th head. In this study, the number of heads is set to 8, and LN (·) represents layer normalization. The function Concat(·) connects the heads together along the channel dimension, and λ is used as the scale parameter in the LN (·) normalization operation.

Finally, to reduce the loss of case fact information caused by network depth in the process of judicial knowledge fusion, we perform a residual connection after a multi-head differential cross attention calculation. The calculation is as follows:


$$\begin{array}{c} v_{f}^{+}=Res\left(v_{f},v_{mda}\right)=(v_{f}+v_{mda})\in R^{n_{f}\times d} \end{array}$$ (20)

where $v_{f}^{+}$ is the case fact feature representation enhanced by judicial knowledge, and Res(•) represents residual connection processing.

### 3.4 Multi-task dependency masking

We input the case fact feature representation $v_{f}^{+}$ enhanced by judicial knowledge into a Long Short-Term Memory (LSTM) network consisting of three time steps, which predicts the law article subtask ${task}_{l}$, charge subtask ${task}_{c}$, and penalty term subtask ${task}_{t}$ in legal judgments, as shown in Fig 2(c). We define the following masked task dependency relationships for these three subtasks: ${task}_{l}≧{task}_{c}$, ${task}_{l}≧{task}_{t}$ and ${task}_{c}≧{task}_{t}$, where the symbol “≥” represents masked task dependency, which only passes the features of the predecessor task to the successor task when the predecessor is predicted to be true during the training phase, otherwise they are not passed on. When pre-task features are successfully transmitted, the post-task relies on pre-task features for prediction.

It is worth noting that our MDMM is a model training mechanism for LJP tasks, which aims to guide model parameters to update in the correct direction. Therefore, MDMM only works during the training phase, and at the inference phase, it assumes that the pre-task prediction is correct, that is, the mask values in subsequent calculation formulas are set to true during the inference phase.

(1) **Using the first-time step in the LSTM network to calculate and predict for the law article subtask** ${task}_{l}$

Firstly, we calculate the decoding hidden state of the law article prediction subtask ${task}_{l}$ as follows:


$$\begin{array}{c} \left[\begin{array}{c} H_{l}\\ Z_{l} \end{array}\right]=LSTMCell\text{1}\left(v_{f}^{+},\left[\begin{array}{c} {\overset{―}{H}}_{l}\\ {\overset{―}{Z}}_{l} \end{array}\right]\right) \end{array}$$ (21)

where  LSTMCell1(·) represents the first time step in the LSTM network, ${\overset{―}{H}}_{l}=\text{0}$ and ${\overset{―}{Z}}_{l}=\text{0}$ respectively represent the initial hidden state and initial memory unit of LSTMCell1, $H_{l}\in R^{d}$represents the decoding hidden state of the law article prediction ${task}_{l}$ obtained by LSTMCell1, $Z_{l}\in R^{d}$ represents the value of the memory unit at the end of the LSTMCell1 processing.

Then, we use a fully connected layer that executes the Softmax function to calculate the predicted results of ${task}_{l}$:


$$\begin{array}{c} \;\;\;\;\;\;\;\;{\hat{y}}_{l}=softmax\left(W_{l}H_{l}+b_{l}\right) \end{array}$$ (22)

$$\begin{array}{c} \;\;\;\;\;\;\;\;\;\;\hat{l}={argmax}_{i_{\text{1}}=\text{1},\cdots ,m}\;({\hat{y}}_{l}(i_{\text{1}}))\; \end{array}$$ (23)

where $W_{l}\in {\text{R}}^{m\times d}$ and $b_{l}\in {\text{R}}^{m}$ are respectively the trainable weight matrix and bias in ${task}_{l}$, ${\hat{y}}_{l}\in {\text{R}}^{m}$ is the probability distribution on the law article labels, $\hat{l}$ is the prediction label of the law article task.

Finally, we determine the mask ${Mask}_{l}$ for subsequent subtasks based on whether the prediction of $\hat{l}$ is correct as follows:


$$\begin{array}{c} {Mask}_{l}=\left\{ \begin{array}{c} True\left(\hat{l}=l_{t}\right)\\ False\left(\hat{l}\neq l_{t}\right) \end{array}\right.\;\; \end{array}$$ (24)

where $l_{t}$ is the true label of the predicted sample in the ${task}_{l}$ task, and ${Mask}_{l}$ is the mask value indicating whether the information of the ${task}_{l}$ task is passed backward.

(2) **Using the second-time step in the LSTM network to calculate and predict for the charge subtask** ${task}_{c}$

Firstly, we apply ${Mask}_{l}$ to the following linear computation to filter out erroneous task dependency information in the ${task}_{l}$ task, receiving only the correct dependency information:


$$\begin{array}{c} \;\;\;\;\;\;\;\;\;\left[\begin{array}{c} {\overset{―}{H}}_{c}\\ {\overset{―}{Z}}_{c} \end{array}\right]=\left(W_{l,c}\left({Mask}_{l}\left[\begin{array}{c} H_{l}\\ Z \end{array}\right]\right)\right)+b_{l,c} \end{array}$$ (25)

where $W_{l,c}\in R^{d\times d}$ and $b_{l,c}\in R^{d}$ are the weight matrix and bias in the linear computation, respectively; ${\overset{―}{H}}_{c}$ and ${\overset{―}{Z}}_{c}$ respectively represent the initial values of hidden states and memory units in the charge subtask ${task}_{c}$.

Then, we use the following formula to calculate the decoding hidden state of the charge subtask ${task}_{c}$:


$$\begin{array}{c} \;\;\;\;\;\;\;\;\;\;\;\left[\begin{array}{c} H_{c}\\ Z_{c} \end{array}\right]=LSTMCell\text{2}\left(v_{f}^{+},\left[\begin{array}{c} {\overset{―}{H}}_{c}\\ {\overset{―}{Z}}_{c} \end{array}\right]\right) \end{array}$$ (26)

where LSTMCell2(·) represents the second time step in the LSTM network, $H_{c}\in R^{d}$ represents the decoding hidden state of the charge subtask ${task}_{c}$ obtained by LSTMCell2, $Z_{c}$ represents the value of the memory unit at the end of the LSTMCell2 processing.

Furthermore, we use the decoding hidden state $H_{c}$ to calculate the prediction results of the charge subtask ${task}_{c}$ in a fully connected layer and obtain the prediction label $\hat{c}$ for ${task}_{c}$.

Finally, we determine the mask ${Mask}_{c}$ for subsequent subtasks based on whether the prediction of $\hat{c}$ is correct:


$$\begin{array}{c} \;\;\;\;\;{Mask}_{c}=\left\{ \begin{array}{c} True\left(\hat{c}=c_{t}\right)\\ False\left(\hat{c}\neq c_{t}\right) \end{array}\right.\; \end{array}$$ (27)

where $c_{t}$ is the true label of the predicted sample in the ${task}_{c}$ task, and ${Mask}_{c}$ is the mask value indicating whether the information of the ${task}_{c}$ task is passed backward.

(3) **Using the third-time step in the LSTM network to calculate and predict for the penalty term subtask** ${task}_{t}$

Firstly, we apply ${Mask}_{l}$ and ${Mask}_{c}$ to the following linear computations to filter out erroneous task dependency information respectively in the ${task}_{l}$ task and the ${task}_{c}$ task, receiving only the correct dependency information:


$$\begin{array}{c} \left[\begin{array}{c} {\overset{―}{H}}_{t}\\ {\overset{―}{Z}}_{t} \end{array}\right]=\left(W_{l,t}\left({Mask}_{l}\left[\begin{array}{c} H_{l}\\ Z_{l} \end{array}\right]\right)\right)+b_{l,t}+\left(W_{c,t}\left({Mask}_{c}\left[\begin{array}{c} H_{c}\\ Z_{c} \end{array}\right]\right)\right)+b_{c,t} \end{array}$$ (28)

where $W_{l,t}\in R^{d\times d}$ and $W_{c,t}\in R^{d\times d}$ are two weight matrices in linear computations, $b_{l,t}\in R^{d}$ and $b_{c,t}\in R^{d}$ are two biases, ${\overset{―}{H}}_{t}$ and ${\overset{―}{Z}}_{t}$ respectively represent the initial values of hidden states and memory units in the penalty term subtask ${task}_{t}$.

Then, we use the following formula to calculate the decoding hidden state of the charge subtask ${task}_{c}$:


$$\begin{array}{c} \left[\begin{array}{c} H_{t}\\ Z_{t} \end{array}\right]=LSTMCell\text{3}\left(v_{f}^{+},\left[\begin{array}{c} {\overset{―}{H}}_{t}\\ {\overset{―}{Z}}_{t} \end{array}\right]\right) \end{array}$$ (29)

where LSTMCell3(·) represents the third time step in the LSTM network, $H_{t}\in R^{d}$ represents the decoding hidden state of the penalty term subtask ${task}_{t}$ obtained by LSTMCell3, $Z_{t}$ represents the value of the memory unit at the end of the LSTMCell3 processing.

Finally, we use a fully connected layer that executes the Softmax function to calculate the predicted results of ${task}_{l}$:


$$\begin{array}{c} {\hat{y}}_{t}=softmax\left(W_{t}H_{t}+b_{t}\right) \end{array}$$ (30)

$$\begin{array}{c} \hat{t}={argmax}_{i_{\text{3}}=\text{1},\cdots ,j}\;({\hat{y}}_{t}(i_{\text{3}})) \end{array}$$ (31)

where $W_{t}\in R^{j\times d}$ and $b_{t}\in R^{j}$ are respectively the trainable weight matrix and bias in ${task}_{t}$, ${\hat{y}}_{t}\in R^{j}$ is the probability distribution on the penalty term labels, $\hat{t}$ is the prediction label of the penalty term task, j is the number of the penalty term labels in the corpus.

### 3.5 Loss function

We calculate the cross-entropy loss for each subtask, and take the loss of all subtasks as the final loss to optimize the model through back propagation:


$$\begin{array}{c} \;L_{JKF-MDM}=-\sum_{x=\text{1}}^{\text{3}}\sum_{z=\text{1}}^{\left|Y_{x}\right|}y_{x,z}log\left({\hat{y}}_{x,z}\right) \end{array}$$ (32)

where$\;\left|Y_{x}\right|$ represents the number of labels for subtask x, $y_{x,z}$ and ${\hat{y}}_{x,z}$ respectively represent the one-hot value and predicted value on label z in subtask x, and $L_{JKF-MDM}$ represents the final loss for the model.

## 4. Experiments

### 4.1 Dataset

The dataset CAIL2018 (https://cail.oss-cn-qingdao.aliyuncs.com/CAIL2018_ALL_DATA.zip) used in this study was originally released as part of the 2018 China Law Research Cup competition. Currently, CAIL2018 has become a large-scale publicly available Chinese legal document dataset that is widely used. Its data sources are highly comprehensive, encompassing up to 5.7 million criminal documents published on the Judicial Documents Network of the Supreme People’s Court of China (https://wenshu.court.gov.cn/). CAIL2018 comprises two sub-datasets, CAIL-small and CAIL-big, each covering case fact descriptions, final judgment articles, charges, and penalty terms.

Consistent with previous research [9,15], the experiments in this study focused on cases involving a single law article and a single charge. We adopted a deletion approach for data in the dataset that lacked the law article, charge, or term-of-penalty labels to ensure the integrity and validity of the data.

The CAIL dataset exhibits a pronounced long-tail distribution in the law article and charge labels: some labels occur extremely infrequently, while a few dominate. To address this issue, we further filtered out the law article and charge labels with a frequency below 100, thereby rationalizing the data distribution. In terms of term-of-penalty prediction, we converted the regression task into a multi – label classification task, carefully defining 11 intervals for classification. Given that the CAIL-big sub-dataset lacked a validation set, we partitioned the processed CAIL-big dataset into training and validation sets at a 9:1 ratio for model training and validation.

In addition, to verify the generalizability of our model in a wider range of legal cases, we followed previous research [9] to build and use a PKU dataset published by Peking University Law Online (http://www.pkulaw.com/). The detailed statistics for the processed dataset are listed in Table 1.

**Table 1:** Statistics of the datasets used.

Data set/ Labels	CAIL-small	CAIL-big	PKU
Training set	101685	1429854	140595
Validation set	13787	159040	17574
Test set	26766	185220	17575
Law article labels	103	118	68
Charge labels	119	130	64
Term-of-penalty labels	11	11	11

### 4.2 Experimental settings

We used the original weights of the pre-trained LERT-base language model and fine-tuned the model during the training phase to adapt to the task of this study. The maximum length of the case-fact input was set to 500. For the training hyperparameters, the batch size (Batch-size) was set to 8, the learning rate to 2e-5, the maximum number of training epochs to 16, and gradient clipping to 5. The AdamW optimizer was adopted to update all the parameters to optimize the model. Additionally, we implemented a learning rate warm-up equivalent to one-tenth of the number of steps per epoch, along with an early stopping mechanism with a patience of 5, to prevent the model from overfitting on the training set. The hyperparameters of the experiments are listed in Table 2.

**Table 2:** Hyper-parameters settings in our model.

Parameter	Setting	Explanation
$n_{A}$	256	Length of the text of law articles
$n_{C}$	150	Length of the text of charges
*λ*	0.2	Hyperparameter in differential attention
max_len	500	Maximum length of case fact input
clip value	5	Gradient clipping
*epochs*	16	Number of iterations
*batch*	8	Batch size
*lr*	2e-5	Learning rate
*dropout*	0.1	Random dropout rate
*early stop*	5	Early stopping epochs

### 4.3 Baselines for comparison

To comprehensively evaluate and analyze the performance of the proposed JKEM model, we compared it with seven SOTA LJP baseline models as follows:

- **FLA** [22] accomplishes the LJP task via an attention-based neural network to fuse relevant legal information.
- **TOPJUDGE** [9] constructs a Directed Acyclic Graph (DAG) based on single topological dependencies among three LJP subtasks for multi-task joint modeling.
- **MPBFN-WCA** [10] develops a multi-view dual-feedback neural network structure, which enables information to undergo multiple interactions and feedback within the network.
- **LANDAN-MTL** [15] constructs a self-learning graph attention network to distinguish confusable law articles and charges, thereby improving the performance of the LJP task.
- **EPM** [14] utilizes event extraction to fuse external legal knowledge with case fact representations, helping the model locate key event information for judgments.
- **GCLA** [16] uses graph contrastive learning and data augmentation techniques to enhance the model’s ability to distinguish different situations.
- **HD-LJP** [11] employs consistency and distinction distillation to model label topological relations among multiple subtasks and improve the differentiation of each subtask itself.
- **Fact-Law Att** [22] uses a neural charge prediction model by capturing the interaction between fact descriptions and applicable laws with attention mechanism.
- **PM** is a pipeline model proposed by TOPJUDGE [9]. It uses three separate CNN classifiers for law articles, charges, and term of penalty. For each subtask, the input is the concatenation of the fact representation and the embeddings for predicted labels of previous subtasks.
- **CNN-MTL** is a conventional multi-task learning method used in TOPJUDGE [9]. It uses CNNs as the fact encoder and does not consider the dependencies among subtasks.
- **HLSTM-MTL** is a conventional multi-task learning method used in TOPJUDGE [9]. It uses hierarchical LSTM networks as the fact encoder and does not consider the dependencies among subtasks.

### 4.4 Comparative results and discussions

Considering that the CAIL2018 dataset suffers from data imbalance, we employed accuracy (Acc), macro-recall (MR), macro-precision (MP), and macro-F1 (MF1) as evaluation metrics. We presented the comparative results of law article predictions on CAIL-small and CAIL-big datasets as shown in Table 3, charge predictions on CAIL-small and CAIL-big datasets as shown in Table 4, and term-of-penalty predictions on CAIL-small and CAIL-big datasets as shown in Table 5, respectively. The comparative results of three sub-task predictions on the PKU dataset are shown in Table 6.

**Table 3:** Comparison of law article predictions on CAIL-small and CAIL-big datasets.

Datasets	CAIL-small				CAIL-big			
Metrics (%)	Acc.	MR	MP	MF_1_	Acc.	MR	MP	MF_1_
FLA	77.72	74.12	75.21	72.78	93.22	64.27	72.81	66.57
TOPJUDGE	78.79	73.39	79.52	73.33	95.81	74.36	84.41	76.67
MPBFN-WCA	79.12	76.02	76.30	74.78	96.06	74.82	85.25	78.36
LANDAN-MTL	81.20	77.38	78.24	76.47	96.57	80.78	86.22	82.36
EPM	**85.65**	78.56	**83.51**	79.76	96.72	79.68	85.79	81.77
GCLA	82.14	77.71	80.08	77.68	96.74	80.82	87.43	82.74
HD-LJP	81.47	78.26	79.63	77.42	96.81	82.08	**88.68**	84.19
Our KFDM	83.12	**82.66**	82.93	**81.75**	**96.94**	**83.70**	87.60	**85.16**

**Table 4:** Comparison of charge predictions on CAIL-small and CAIL-big datasets.

Datasets	CAIL-small				CAIL-big			
Metrics (%)	Acc.	MR	MP	MF_1_	Acc.	MR	MP	MF_1_
FLA	80.98	77.92	79.11	76.77	92.48	68.12	76.21	69.97
TOPJUDGE	82.03	79.33	83.14	79.03	95.73	79.49	87.99	81.93
MPBFN-WCA	82.14	80.72	82.28	80.72	95.98	79.73	89.16	83.20
LANDAN-MTL	85.07	82.52	83.42	82.74	96.45	83.73	88.51	85.35
EPM	85.39	80.74	85.54	82.16	96.45	81.93	88.78	82.84
GCLA	86.08	82.58	85.41	82.99	96.63	83.75	89.74	85.87
HD-LJP	87.41	84.56	86.08	84.72	96.64	84.34	90.41	86.35
Our KFDM	**89.15**	**86.45**	**87.11**	**86.28**	**97.80**	**87.26**	**92.30**	**88.96**

**Table 5:** Comparison of term-of-penalty predictions on CAIL-small and CAIL-big datasets.

Datasets	CAIL-small				CAIL-big			
Metrics (%)	Acc.	MR	MP	MF_1_	Acc.	MR	MP	MF_1_
FLA	36.32	28.22	30.81	27.83	57.66	38.89	43.01	41.63
TOPJUDGE	36.05	32.49	34.54	29.19	57.29	42.61	47.35	44.03
MPBFN-WCA	36.02	28.60	31.94	29.85	58.14	39.07	45.86	41.30
LANDAN-MTL	38.29	32.49	36.16	32.65	59.66	45.34	51.78	46.93
EPM	37.59	32.51	35.32	33.14	58.67	45.86	53.93	46.58
GCLA	37.03	29.86	33.80	29.88	56.13	43.36	47.87	44.65
HD-LJP	**42.46**	36.67	40.20	37.07	60.35	50.04	52.66	50.17
Our KFDM	42.39	**37.34**	**40.98**	**38.09**	**62.17**	**50.10**	**54.29**	**51.82**

**Table 6:** Comparison of three sub-task predictions on PKU dataset.

Tasks	Law Articles(%)				Charges(%)				Term of Penalty(%)			
Metrics	Acc.	MR	MP	MF_1_	Acc.	MR	MP	MF_1_	Acc.	MR	MP	MF_1_
Fact-Law Att	93.9	68.1	63.4	63.5	94.2	65.8	58.5	58.7	55.7	27.7	27.4	26.5
PM	94.4	69.6	61.0	62.2	94.3	65.1	56.2	57.2	58.2	36.2	26.4	27.1
CNN-MTL	95.0	73.8	64.9	66.0	95.0	70.7	60.6	61.7	58.4	36.0	28.7	28.9
HLSTM-MTL	93.9	71.2	64.6	65.1	93.8	67.8	60.0	60.7	55.4	31.3	26.2	25.7
TOPJUDGE	95.4	76.4	67.6	68.4	95.6	75.9	69.6	70.9	57.8	38.9	32.1	31.8
Our KFDM	**97.6**	**86.1**	**70.5**	**76.3**	**97.9**	**82.7**	**72.9**	**77.4**	**68.0**	**44.7**	**38.1**	**41.5**

Based on the above comparative results, we can draw the following conclusions. First, our KFDM model achieved the best performance in terms of MF1 in the law article prediction task on both datasets. This indicates that by introducing two types of external judicial knowledge—law articles and charge explanations—and integrating them into case fact representation vectors, this study can better highlight key case element information in case facts. Meanwhile, this also proves that the multi-head differential attention mechanism can achieve a deep fusion of judicial domain knowledge and case facts, and significantly strengthen the representation vectors of case facts.

Second, on both the CAIL-small and CAIL-big datasets, our KFDM model achieved a substantial improvement in the charge prediction task compared with the baseline models. There are two key reasons for this. First, the introduction of the aforementioned two types of external judicial knowledge (law articles and charge explanations) enhances the semantic representation of case fact vectors. This enables the model to more accurately capture case features, thereby improving the accuracy of charge prediction. Second, the multi-task dependency masking strategy proposed in this study effectively masks the erroneous task dependency information transmitted from the prepositive law article prediction task to the charge prediction task, avoiding prediction biases caused by interference from such erroneous task dependency information.

Finally, in the most challenging sentence prediction task, the overall performance of our KFDM model on both the CAIL-small and CAIL-big datasets outperformed all baseline models, and its MF1 value was further improved. These excellent results are attributed to the following: the texts of law articles contain clear and explicit definitions of the applicable sentence ranges for each charge. Thus, by integrating external judicial knowledge into case facts, our KFDM model enriches these facts with information about applicable statutory sentence ranges derived from law articles. This enables the model to precisely capture the target statutory sentence ranges corresponding to different charges. Furthermore, the multi-task dependency masking strategy also masks the erroneous task dependency information from the prepositive law article and charge prediction tasks, thereby allowing the term-of-penalty prediction to be free from interference from prepositive erroneous task dependency information.

Furthermore, the results in Table 6 show that our KFDM model significantly outperforms the five baselines in three subtasks of the niche PKU dataset. This demonstrates that our KFDM model also possesses very strong generalizability in more diverse legal cases.

### 4.5 Ablation study

To verify the effectiveness of the proposed method, we conducted the following four ablation comparison experiments on the CAIL-small dataset:

- **w/o JKFM**: removes the judicial knowledge extraction module and judicial knowledge fusion module judicial knowledge fusion module in our KFDM.
- **w/o MDMM**: removes the multi-task dependency masking module in our KFDM.
- w/o CNN: removes convolutional neural networks from knowledge extraction in KFDM.
- **JKF+TOPJD**: based on the legal knowledge extraction module of our KFDM, replaces the multi-task dependency masking module (MDMM) with TOPJUDGE proposed by Zhong et al. [9].
- **LERT**: removes all modules from our KFDM and directly encodes case facts using LERT.

Table 7 presents the results of the ablation experiments on our KFDM model using the CAIL-small dataset.

**Table 7:** Results of ablation comparison experiments on the CAIL-small dataset.

Tasks	Law Articles(%)				Charges(%)				Term of Penalty(%)			
Metrics	Acc.	MR	MP	MF_1_	Acc.	MR	MP	MF_1_	Acc.	MR	MP	MF_1_
Our KFDM	**83.12**	**82.66**	**82.93**	**81.75**	**89.15**	**86.45**	**87.11**	**86.28**	**42.39**	**37.34**	**40.98**	**38.09**
LERT	82.02	79.23	81.68	79.19	85.07	82.47	84.88	82.93	39.21	30.49	37.02	31.37
JKF+TOPJD	82.80	82.16	82.44	81.29	86.88	85.89	86.10	85.60	41.06	36.01	40.43	37.50
w/o JKFM	82.49	81.33	81.67	80.77	87.59	86.02	86.56	85.74	40.53	35.05	40.46	36.29
w/o MDMM	82.20	81.57	81.68	81.10	86.49	84.16	85.51	85.19	41.16	34.54	39.46	35.58
w/o CNN	82.93	82.31	82.63	81.45	88.82	86.29	86.86	86.12	42.09	37.14	40.43	37.90

Through an analysis of these results, several conclusions can be drawn. First, our KFDM model significantly outperforms the w/o JKFM model (with the judicial knowledge fusion module removed) across all evaluation metrics. The MF1 value of the w/o JKF model in the law article prediction task decreased by 0.98% compared with that of the KFMD model, which indicates that the introduction of external judicial knowledge can improve the accuracy of the law article prediction task. Meanwhile, the multi-task modeling paradigm relying on the multi-task dependency masking strategy can still achieve good performance in the law article prediction task. In addition, the MF1 values in the charge prediction and term-of-penalty prediction tasks show a certain decrease because subsequent tasks cannot learn the corresponding judicial knowledge for these tasks from law articles and charge interpretation knowledge.

Second, our KFDM model significantly outperforms the w/o MDM model (with the multi-task dependency masking strategy module removed) across all evaluation metrics. The MF1 values of the w/o MDMM model in the three subtasks are lower than those of the KFDM model; specifically, in the charge prediction and term-of-penalty prediction tasks, its MF1 values drop by 1.09% and 2.51%, respectively, compared with the KFDM model. This indicates that removing the multi-task dependency masking strategy module will lead to the transmission of erroneous task dependency information between subtasks, thereby reducing the prediction accuracy of subsequent subtasks.

Third, the JKF+TOPJUDGE model uses the TOPJUDGE method proposed by Zhong et al. [9] to replace the MDMM module. It can be seen from the experimental results that the TOPJUDGE method performs subtask dependency modeling by constructing a DAG, but fails to consider the issue of erroneous task dependency information transmission between subtasks, resulting in a decline in its performance.

Fourth, when only the LERT pre-trained language model is used for the LJP task, its performance across all metrics is lower than that of KFDM, JKF+TOPJUDGE, w/o JKFM, and w/o MDMM. This indicates that all the methods and strategies proposed in this study are effective and feasible.

Fifthly, after removing the CNN module in knowledge extraction, the performance of the model decreases to varying degrees in three subtasks. Firstly, law article texts (such as the definition of theft in Article 264 of the Criminal Law) contain structured information of “constituent elements + sentencing standards”, and CNN’s local semantics can accurately capture key local features such as “large amount” and “imprisonment for less than three years”. When CNN is absent, the local features extracted by LERT are insufficient, leading to a decline in the accuracy of law article matching. Furthermore, charge explanation texts focus more on “behavior definition + distinction from similar charges”, e.g., the behavioral differences between theft and robbery; and CNN can strengthen such fine-grained local semantics. When CNN is absent, the feature discrimination is weakened, making charge prediction prone to confusing similar charges. Finally, term-of-penalty prediction relies on legal provision sentencing ranges, basic penalties corresponding to charges, and case circumstance matching simultaneously; and the judicial knowledge features optimized by CNN serve as the key bridge connecting these three aspects. When CNN is absent, the effectiveness of knowledge fusion decreases, leading to an increase in the deviation of term-of-penalty range prediction.

### 4.6 Hyperparameter study

In terms of the impact of hyperparameters on model performance, we conducted three supplementary experiments to verify the effect of different combinations of hyperparameters on the experimental results. The hyperparameter combinations used are shown in Table 8, and the experimental results are shown in Table 9:

**Table 8:** Hyperparameter combinations.

Parameter	KFDM Setting	Refer1	Refer2	Refer3
$n_{A}$	256	256	256	156
$n_{C}$	150	150	150	100
*λ*	0.2	0.3	0.2	0.2
max_len	500	400	500	500
clip value	5	5	5	5
*epochs*	16	16	8	16
*batch*	8	8	8	8
*lr*	2e-5	2e-5	1e-5	3e-5
*dropout*	0.1	0.1	0.1	0.1
*early stop*	5	5	5	5

**Table 9:** Results of Hyperparameter comparison experiments on the CAIL-small dataset.

Tasks	Law Articles(%)				Charges(%)				Term of Penalty(%)			
Metrics	Acc.	MR	MP	MF_1_	Acc.	MR	MP	MF_1_	Acc.	MR	MP	MF_1_
KFDM	**83.12**	**82.66**	**82.93**	**81.75**	**89.15**	**86.45**	**87.11**	**86.28**	**42.39**	**37.34**	**40.98**	**38.09**
Refer1	82.86	82.23	82.77	81.39	88.78	86.15	86.93	86.03	42.12	37.01	40.55	37.83
Refer2	82.46	81.89	82.03	80.77	88.24	85.71	86.97	85.47	39.64	36.93	39.67	36.81
Refer3	82.65	82.32	82.40	81.09	88.47	85.99	86.73	85.88	39.92	36.88	40.33	37.17

The results of hyperparameter experiments in Table 9 can draw the following conclusions:

Refer1: Reducing the length of case facts truncates some descriptive texts that may play a key role in case facts, which has a certain impact on the integrity of semantic representation. Meanwhile, increasing hyperparameter *λ* in differential attention slightly raises the differential attention weights, which may prevent some external judicial knowledge information from being integrated, thus failing to achieve optimal performance.
Refer2: Reducing the number of epochs and lowering the learning rate *lr* slow down the parameter update speed, which only slightly delays the training convergence process. Therefore, optimal performance cannot be achieved with fewer epochs.
Refer3: Reducing the length of legal texts may truncate some key judicial knowledge information in law articles and charge explanations, thus leading to the loss of information about core constituent elements and sentencing criteria. Meanwhile, increasing the learning rate enlarges the magnitude of parameter updates, which in turn results in deviations in the training and prediction direction of the overall model.

### 4.7 Masking rate and case analysis in MDMM

**Fig 4:**
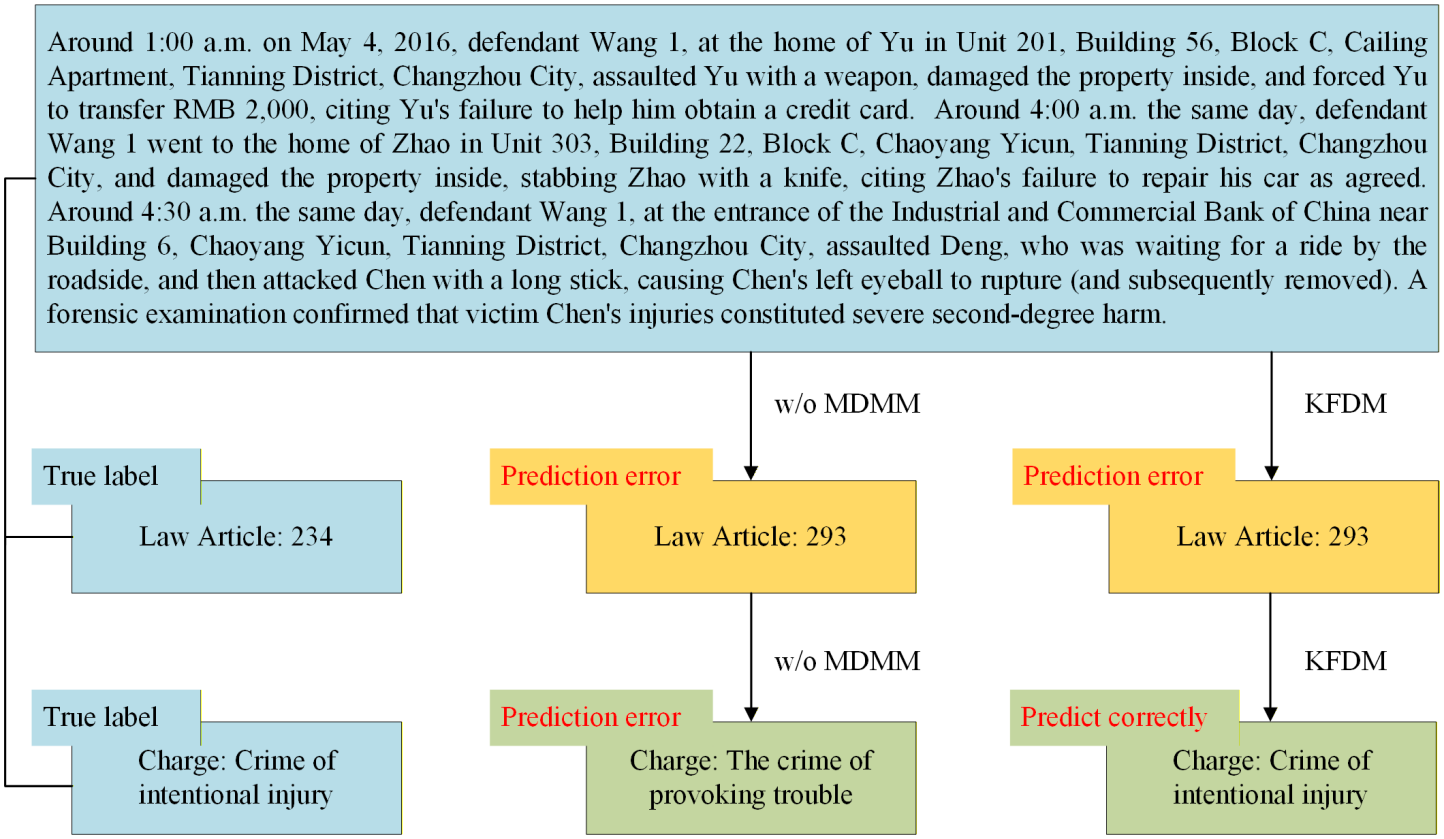
Case analysis for MDMM.

To reveal how our MDMM works, we take the dependency relation ${task}_{l}≧{task}_{c}$ as an example, and calculated the masking rate during the model training phase on the CAIL-small dataset, as shown in Table 10. In addition, we also provided a complex case where the dependency masking mechanism works, as shown in Fig 4.

**Table 10:** Masking rate of each epoch on the CAIL-small training set.

Epoch	Error law article predictions	Masking rate	Epoch	Error law article predictions	Masking rate
*Epoch1*	50700	49.86%	*Epoch9*	19879	19.55%
*Epoch2*	37806	37.18%	*Epoch10*	19157	18.84%
*Epoch3*	30373	29.87%	*Epoch11*	18527	18.22%
*Epoch4*	27780	27.32%	*Epoch12*	18141	17.84%
*Epoch5*	25696	25.27%	*Epoch13*	17775	17.48%
*Epoch6*	23876	23.48%	*Epoch14*	17490	17.20%
*Epoch7*	22350	21.98%	*Epoch15*	17307	17.02%
*Epoch8*	20978	20.63%	*Epoch16*	17164	16.88%

The results in Table 10 reveal that: For dependency relation ${task}_{l}≧{task}_{c}$, the masking rate is high in the initial few epochs due to the low accuracy of the model and the large number of incorrectly predicted law articles. In the later epochs, as the model accuracy improves and the number of samples with incorrect law article predictions decreases, the masking rate decreases but remains above 16%. Therefore, the results in Table 10 demonstrate that our dependency masking mechanism plays an important role throughout the entire training phase of the model.

Fig 4 shows a case sample with a very complex crime: the defendant Wang 1 committed crimes at three different locations on the same day. At the first location, he simultaneously committed assault, property damage, and robbery. However, the crimes committed at all three locations were primarily intentional assault. Due to the complexity of the case, the law article prediction for this sample was incorrect. In the w/o MDMM model, the lack of dependency masking led to an incorrect charge prediction. However, in our KFMD model, because MDMM masked the incorrect law article dependency information, the charge was correctly predicted. This case analysis demonstrates that our MDMM can effectively improve the performance of the dependency-based LJP method in the subsequent tasks of samples with complex circumstances.

## 5. Conclusions

In this paper, we propose a novel legal judgment prediction model based on knowledge fusion and dependency masking, aiming to deeply understand and utilize external judicial knowledge while filtering out erroneous information in multi-task dependencies. Through experimental evaluation, this study revealed the following: (1) Incorporating a CNN-based local semantic refinement component on top of the pre-trained language model enables further extraction of the core knowledge embedded in judicial documents; (2) introducing differential attention can reduce noise in the fusion process of external legal knowledge and case facts, and more accurately locate key information in case facts; and (3) introducing a multi-task dependency information masking mechanism enables accurate identification and filtering of erroneous dependency information, thereby further improving the model’s performance.
